# Recurrent tuberculosis in the Netherlands – a 24-year follow-up study, 1993 to 2016

**DOI:** 10.2807/1560-7917.ES.2022.27.12.2100183

**Published:** 2022-03-24

**Authors:** Connie Erkens, Betül Tekeli, Dick van Soolingen, Henrieke Schimmel, Suzanne Verver

**Affiliations:** 1 KNCV Tuberculosis Foundation, The Hague, the Netherlands; 2 Vrije Universiteit Amsterdam, Amsterdam, the Netherlands; 3 National Institute for Health and Environment (RIVM), Bilthoven, the Netherlands; 4 Department of Public Health, Erasmus MC, Rotterdam, the Netherlands

**Keywords:** recurrent TB, tuberculosis, reactivation, reinfection

## Abstract

**Background:**

Not all treated tuberculosis (TB) patients achieve long-term recovery and reactivation rates reflect effectiveness of TB treatment.

**Aim:**

We aimed to estimate rates and risk factors of TB reactivation and reinfection in patients treated in the Netherlands, after completed or interrupted treatment.

**Methods:**

Retrospective cohort study of TB patients with available DNA fingerprint data, registered in the Netherlands Tuberculosis register (NTR) between 1993 and 2016. Reactivation was defined as an identical, and reinfection as a non-identical *Mycobacterium tuberculosis* strain in sequential episodes.

**Results:**

Reactivation rate was 55/100,000 person-years (py) for patients who completed, and 318/100,000 py for patients who interrupted treatment. The risk of reactivation was highest in the first 5 years after treatment in both groups. The incidence rate of reactivation was 228/100,000 py in the first 2 years and 57/100,000 py 2–5 years after completed treatment. The overall rate of reinfection was 16/100,000 py. Among those who completed treatment, patients with male sex, mono or poly rifampicin-resistant TB and a previous TB episode had significantly higher risk of reactivation. Extrapulmonary TB was associated with a lower risk. Among patients who interrupted treatment, directly observed treatment (DOT) and being an undocumented migrant or people experiencing homelessness were associated with a higher risk of reactivation.

**Conclusions:**

Both patients who completed or interrupted TB treatment should be considered as risk groups for reactivation for at least 2–5 years after treatment. They patients should be monitored and guidelines should be in place to enhance early detection of recurrent TB.

## Introduction

Globally, there is an increase in treatment success rates of tuberculosis (TB), with an estimated 83% treatment success of new patients in 2016 [1]. Treatment success is defined as the proportion of patients successfully treated or cured and is a proxy for the effectiveness of TB treatment. Unfortunately, not all TB patients treated successfully achieve long-term recovery [2]. Some patients may experience a re-occurrence of the disease, which is referred to as recurrent TB and can be caused by either a reactivation or a reinfection [2-4]. The rate of reactivated TB can be considered as a more accurate indicator for the quality and effectiveness of TB treatment and care on an operational level than treatment success.

DNA fingerprinting of the bacterial isolates has enabled the differentiation between recurrent TB in endogenous reactivation and exogenous reinfection [5,6]. Reactivation is defined as re-inflammation with an identical *Mycobacterium tuberculosis* strain as isolated in the initial episode of the disease [3,5]. Reinfection is the infection with a different *M. tuberculosis* strain than isolated in the previous episode(s) of the disease [3,5]. Higher rates of reactivation and reinfection were reported in countries with a high incidence of TB compared with countries with a low TB incidence. To illustrate, although with different time frames for follow-up, reactivation rates of 2.7–4.7% have been reported in countries with a high TB incidence compared with 0.3–1.0% in countries with a low TB incidence [6-9]. Likewise, higher reinfection rates of 1.5–2.8% have been found in countries with a high incidence of TB compared with 0.1–0.3% in countries with a low incidence of TB [4,8-20].

Even though risk factors for reactivation of TB in countries with a low TB incidence have been described, previous studies have had limited power to explore the findings over an extended time period. Additionally, only a few studies explored the effect of interrupted treatment on the risk of reactivation [6,13].

The aim of this study was to estimate the rate of reactivation and reinfection of TB in patients diagnosed and treated in the Netherlands in the period 1993–2016 after treatment completion and treatment interruption. We used data of more than 16,000 notified patients with culture-positive TB. Additionally, risk factors for reactivation and reinfection among patients who completed the treatment were analysed.

## Methods

### Study design and setting

Data for this retrospective cohort study of TB patients notified during the period 1993–2016 were obtained from the database of the Netherlands Tuberculosis register (NTR) [21]. We retrieved demographic data (sex, age, date of birth, country of birth, year of entry in the Netherlands), previous TB, year of diagnosis of previous TB episode, type of TB drug resistance, bacterial culture results, DNA fingerprint results, risk factors for TB, HIV status and other co-morbidities, treatment regimen, treatment outcome and duration, and directly observed treatment (DOT). The variables for previous TB and treatment outcome were defined according to recommendation of the European Centre for Disease Prevention and Control (ECDC) and the World Health Organization (WHO) [22]. Patients with drug-susceptible TB were treated with a standardised regimen according to WHO recommendations. DOT was used in patients with a perceived high risk of non-adherence or drug resistance [23].

### DNA fingerprinting

Since 1993, the National Institute for Health and Environment (RIVM) performs nationwide DNA fingerprinting of *M. tuberculosis* positive isolates. Until 2009, restriction fragment length polymorphism (RLFP) was used to identify the specific insertion sequences IS*6110*. From 2009 onwards, the 24-locus variable number of tandem repeats (VNTR) has been used. Isolates from 2004 until 2009 were re-typed retrospectively by the VNTR method. The analysis for both IS*6110* RFLP and VNTR typing was conducted using Bionumerics software, version 5.0 (Applied Maths, Saint-Martens-Latem, Belgium).

### Definitions

In the Netherlands, a patient with bacteriologically-confirmed TB or with a clinical diagnosis requiring TB treatment is notified to the NTR. Patients with recurrent TB more than 2 months after the end of a previous treatment are registered as previously treated patients. We defined recurrent TB as a new episode of active TB in a patient, 2 months or longer after completing the treatment of the first recorded episode in the database, either due to reactivation or reinfection. For this study, reactivation was defined as two TB episodes caused by an identical *M. tuberculosis* strain. A reinfection was defined as two TB episodes caused by different *M. tuberculosis* strains. For the main analysis, fingerprints with one or more bands difference in the RFLP or one or more repeats difference in the VNTR were considered as different *M. tuberculosis* strains signifying reinfection. For the sensitivity analysis, fingerprints with more than two differences in bands in the RFLP or in repeats in the VNTR were considered as different *M. tuberculosis* strains, while differences of up to two bands/repeats were considered reactivation. The follow-up period for patients with a recurrence was the time between the date of the end of treatment of the first episode of the disease until the date of diagnosis of the second episode. For patients without a recurrence, the follow-up period was the time between the end of treatment until 31 December 2016 expressed in the number of person-years.

### Study population

Data on all culture-positive TB patients with a known DNA fingerprint pattern registered from 1993 until 2016 in the NTR were included. To identify patients with recurrent TB episodes, we traced potential duplicate cases in the NTR by sex, date of birth, country of birth, year of diagnosis, previous TB episode and year of entry in the Netherlands. For patients reported after 2005, the NTR record number of the previous episode was registered in the NTR database and could also be used for matching. In addition, for patients reported after 2010, recurrent episodes were recorded in the RIVM laboratory data system, which allowed us to match recurrent entries with NTR data. For all potential duplicate cases before 2005, RIVM experts checked if the RIVM laboratory numbers recorded in the NTR database applied to the same individual in the RIVM laboratory register.

For the cohort analysis, we excluded patients with a treatment failure, patients who completed their treatment after 31 December 2016, patients with an unknown treatment outcome or unknown treatment duration, patients who continued treatment outside the Netherlands, patients who died and all patients with multi-drug resistant TB or resistance against pyrazinamide or ethambutol.

### Statistical analysis

We calculated reactivation and reinfection rates from the number of recurrent cases identified per 100,000 person-years followed-up (py), stratified according to treatment outcome i.e. completed treatment vs interrupted treatment. We calculated incidence rates for the first 2 years of follow-up, for 2–5 years of follow-up, and for > 5 years of follow-up. We performed univariate and multivariable Cox regression analysis to determine risk factors for reactivation and reinfection and calculated the unadjusted hazard ratios (HR) of potential risk factors. We performed a sensitivity analysis with the alternative definition of reactivation and reinfection. Characteristics considered as potential risk factors for reactivation and/or reinfection were: sex, age group, incidence in country of birth, DOT, type of TB, drug resistance, alcohol or drug use, experiencing homelessness or being an undocumented migrant, HIV or other comorbidities (such as diabetes mellitus), immunosuppressive medication (such as tumor necrosis factor (TNF)-alpha blockers use), previous TB treatment, TB treatment regimen, TB treatment outcome, treatment duration and adverse events following TB medication. We performed a stratified analysis of risk for reactivation among patients who completed the treatment and among those who interrupted the treatment. Adjusted HR were calculated for all variables with a p value of ≤ 0.20 in the univariate analysis. We used a stepwise backward elimination method for a multivariable model. In the final model, we considered risk factors with a p value of < 0.05 statistically significant and performed in SPSS version 25 for Mac (Chicago, United States). Mid-P exact test to calculate incidence rates and the 95% confidence interval (CI) and Chi-square test to compare rates were performed in Open Epi 3.01.

### Ethical approval

Permission to use the NTR data for this study was obtained from the NTR registration committee. The second step in the matching process to verify the potential duplicate cases was done by the NTR data manager at RIVM, who is authorised to access person-identifiable data. Verification of the RIVM laboratory numbers in the RIVM laboratory register was done by authorised RIVM laboratory staff. Only anonymised data were provided to the researchers for the analysis. In the Netherlands, ethical approval is not needed for the use of anonymised data from the NTR.

## Results

### Description of patients

A total of 30,273 TB patients were notified in the NTR between 1993 and 2016, of whom 22,437 (74.1%) had culture-confirmed TB, and of whom 18,886 (84.2%) had a known DNA fingerprint result. The following patients were excluded from the study: 1,492 (7.9%) patients who died, 592 (3.1%) who continued their treatment elsewhere, 359 (1.9%) patients with multidrug-resistant TB or primary resistance against pyrazinamide or ethambutol, and 473 (2.5%) patients with an unknown treatment duration, unknown sex or a follow-up time of less than 0.17 years (2 months) after the end of treatment. A total of 15,970 patients were included in the analysis, of whom 15,136 (94.8%) patients completed their treatment successfully and 834 (5.2%) interrupted their treatment. The characteristics of the included patients are described in Table 1.

**Table 1:** Characteristics during the first episode of the disease of all confirmed culture-positive tuberculosis patients with an available DNA fingerprint in both episodes, the Netherlands, 1993 to 2016 (n = 15,970)

Characteristics	Level	Total cohort		Reactivation		Reinfection	
		n	Column % ^a^	n	Row % ^a^	n	Row % ^a^
**Total**		**15,970**	**100**	**141**	**0.9%**	**31**	**0.2%**
Year of diagnosis	1993–98	4,551	28%	42	0.9%	10	0.2%
	1999–2004	4,747	30%	40	0.8%	11	0.2%
	2005–10	4,026	25%	41	1.0%	10	0.2%
	2011–16	3,130	20%	18	0.6%	0	0.0%
Age group	0–14	478	3%	4	0.8%	2	0.4%
	15–24	3,290	21%	23	0.7%	9	0.3%
	25–34	4,544	28%	30	0.7%	9	0.2%
	35–44	2,856	18%	33	1.2%	5	0.2%
	45–54	1,854	12%	17	0.9%	4	0.2%
	55–64	1,283	8%	13	1.0%	2	0.2%
	65 +	2,149	13%	21	1.0%	0	0.0%
Age	Median (Q1, Q3)	34	25–50	39	26–53	29	21–42
Sex^b^	Male	9,803	61%	101	1.0%	22	0.2%
	Female	6,648	42%	40	0.6%	9	0.1%
Treatment duration in days	Median (Q1, Q3)	196	183–273	185	167–242	193	182–280
Treatment outcome	Completed treatment successfully	15,136	95%	102	0.7%	26	0.2%
	Interrupted treatment	834	5%	39	4.7%	5	0.6%
	*0–3 months treatment*	*305*	*2%*	*16*	*5.2%*	*1*	*0.3%*
	*4–6 months treatment*	*291*	*2%*	*19*	*6.5%*	*2*	*0.7%*
	*≥ 7 months treatment*	*238*	*1%*	*4*	*1.7%*	*2*	*0.8%*
DOT	Yes	3,140	20%	40	1.3%	8	0.3%
	No / unknown	12,830	80%	101	0.8%	23	0.2%
Type TB	Sputum AFB pos PTB	5,105	32%	61	1.2%	17	0.3%
	BAL AFB pos PTB or AFB neg cavernous PTB	1,151	7%	14	1.2%	2	0.2%
	AFB neg and culture pos PTB	4,292	27%	39	0.9%	9	0.2%
	ETB	5,422	34%	27	0.5%	3	0.1%
Drug resistance	(Probably) susceptible^c^	14,905	93%	130	0.9%	28	0.2%
	Mono / poly H	1,026	6%	9	0.9%	2	0.2%
	Mono / poly R	39	0%	2	5.1%	1	2.6%
Homelessness or being undocumented	No	13,666	86%	106	0.8%	21	0.2%
	Yes	2,304	14%	35	1.5%	10	0.4%
Alcohol or drug use	No	15,479	97%	129	0.8%	28	0.2%
	Yes	663	4%	12	1.8%	3	0.5%
Comorbidity	No comorbidity or unknown	14,036	88%	114	0.8%	28	0.2%
	Comorbidity^d^	1,266	8%	16	1.3%	0	0.0%
	HIV	668	4%	11	1.6%	3	0.4%
Previous TB episode	No	15,383	96%	126	0.8%	31	0.2%
	Yes	587	4%	15	2.6%	0	0.0%
Country of birth	The Netherlands	4,731	30%	46	1.0%	3	0.1%
	Outside the Netherlands	11,239	70%	95	0.8%	28	0.2%
TB incidence in country of origin of migrants	< 100 per 100,000	4,180	26%	39	0.9%	13	0.3%
	100–200 per 100,000	2,581	16%	27	1.0%	3	0.1%
	> 200 per 100,000	4,312	27%	29	0.7%	12	0.3%
	Unknown	166	1%	0	0.0%	0	0.0%

AFB: acid-fast bacilli; BAL: broncho-alveolar lavage; DOT: directly observed treatment; E: ethambutol; ETB: extrapulmonary TB; MDR TB: multidrug-resistant TB; mono/poly H: mono-/poly-resistance against isoniazid other than MDR TB; mono/poly R: mono-/poly-resistance against rifampicin other than MDR TB; neg: negative;n: number of patients; pos: positive; PTB: pulmonary TB; Q1: first quartile; Q3: third quartile; sec: second; standard regimen: 2HRZ(E)/6HR(E).

^a^ The percentages shown are column percentages for the cohort and row percentages for reactivation and reinfection. For continuous variables, interquartile range (Q1-Q3) is indicated.

^b^ Three patients with unknown sex (excluded in the multivariable analysis).

^c^ Until 2005, drug sensitivity was only recorded when drug resistance was detected, hence all cases without registered drug resistance were categorised as “probably susceptible “.

^d^ Comorbidity such as diabetes, malnutrition, malignancy or use of immunosuppressive medication.

### Incidences

Using the main definition of reactivation as infection with a strain with an identical DNA fingerprint, a total of 141 (0.9%) patients were identified with a reactivation and 31 (0.2%) with a reinfection. Five patients had a recurrent infection with a strain with one or two differences in the DNA fingerprint. The risk of reactivation (or rate ratio) was 5.8 times higher (95% confidence interval (CI): 4.0–8.4) in patients who interrupted their treatment (incidence: 318/100,000 py,) compared with patients who completed their treatment successfully in the first episode of the disease (reactivation rate: 55/100,000 py). The reinfection rate (16/100,000 py) was lower than the overall rate of reactivation (71/100,000 py). The median time to reactivation was 1.1 years and to reinfection was 5.5 years. (Supplement Table S1 shows the incidence rates for reactivation and reinfection).

**Figure 1:**
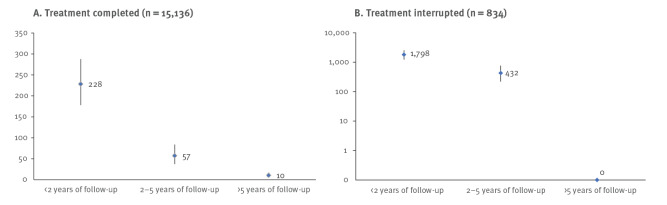
Tuberculosis incidence rates of reactivation per 100,000 person-years in the first 2 years, 2−5 years and more than 5 years after treatment among (A) those who completed treatment and (B) those who interrupted treatment, the Netherlands, 1993 to 2016

The highest incidence of reactivation was in the first 2 years after finishing treatment, both for patients who completed or interrupted their treatment. In that period, the incidence of reactivation was 228 per 100,000 py (95% CI: 178–288) for patients who completed their treatment and 1,798 per 100,000 py (95% CI: 1,227–2,548) for patients who interrupted their treatment. After 2−5 years after treatment, the incidence remained high for both patients who completed their treatment in the first episode of the disease (incidence: 57/100,000 py; 95% CI: 37–84) and those who interrupted their treatment (incidence: 432/100,000 py; 95% CI: 219–769) (Figure 1 and Supplement Table S2 show incidence rates by duration of follow-up).

Bars indicate the 95% confidence interval. Underlying data are in the Supplement Table S2.

### Risk factors for reactivation

Table 2 shows the risk factors for reactivation in patients who completed their treatment in the first episode of disease. In the multivariable analysis, males (adjusted (a) HR: 1.9; 95% CI: 1.2–2.9), patients with mono or poly rifampicin (R)-resistant TB (aHR: 6.6; 95% CI: 1.6–27.3) and patients with a previous TB episode before 1993 or outside the Netherlands (aHR: 3.2; 95% CI: 1.7–5.9) had a significant higher risk of reactivation. Extrapulmonary TB was associated with a lower risk of reactivation (aHR: 0.5; 95% CI: 0.3–0.9). Patients with comorbidity or who were HIV-positive also had a higher risk for reactivation, but the p value of the aHR was more than 0.05.

For patients who interrupted their treatment, those receiving DOT (aHR: 2.2; 95% CI: 1.1–4.3) and migrants who were undocumented or people experiencing homelessness (aHR: 2.1; 95% CI: 1.1–4.1) were associated with a higher risk for reactivation (Supplement Table S3 displays the risk factors for reactivation among interrupters). Receiving treatment for 7 or more months (aHR: 0.3; 95% CI: 0.1–0.9) was associated with a lower risk for reactivation. Extrapulmonary TB (aHR: 0.4; 95% CI: 0.1–1.0) was not significantly (p = 0.06) associated with a lower risk for reactivation (Supplement Table S3).

In the sensitivity analysis with reactivation defined as an infection with a strain with > 2 differences in the DNA fingerprint, patients who were HIV-positive also had a significant higher risk for reactivation among patients who completed the TB treatment (Supplement Table S4 displays risk factors for reactivation after treatment completion (reactivation <2 differences in the DNA fingerprint)). The other associations remained the same.

**Table 2:** Risk factors^a^ associated with a reactivation for patients who completed their treatment successfully, the Netherlands, 1993 to 2016 (n = 15,136)

Category	Level	Cohort n	Person-years follow-up	Events n	Incidence per 100,000 py (95% CI)	Univariate analysis			Multivariable analysis		
						Crude HR	95% CI	p value	aHR^b^	95% CI	p value
Sex^c^	Female	6,162	76,198	26	34 (23–49)	Ref	NA	NA	Ref	NA	NA
	Male	8,971	110,376	76	69 (55–86)	2.0	1.3–3.2	0.00	**1.9**	**1.2–2.9**	**0.01**
Treatment regimen	Standard regimen	11,086	164,381	83	50 (40–62)	Ref	NA	NA	Ref	NA	NA
	Other	3,874	19,834	14	71 (40–116)	0.6	0.4–1.1	0.13	0.6	0.3–1.1	0.10
	Unknown	176	2,397	5	209 (76–462)	3.8	1.6–9.5	0.00	**4.0**	**1.6–9.9**	**0.00**
Age group	0–14	447	5,922	4	68 (21–163)	1.0	0.3–3.0	0.97	NA	NA	NA
	15–24	2,986	37,966	16	42 (25–68)	0.6	0.3–1.2	0.16	NA	NA	NA
	25–34	4,165	52,366	19	36 (22–56)	0.5	0.3–1.0	0.06	NA	NA	NA
	35–44	2,630	31,667	21	66 (42–100)	0.9	0.5–1.8	0.82	NA	NA	NA
	45–54	1,743	20,092	15	75 (43–120)	1.0	0.5–2.0	0.97	NA	NA	NA
	55–64	1,202	14,147	10	71 (36–126)	1.0	0.4–2.1	0.93	NA	NA	NA
	65 +	1,963	24,452	17	70 (42–109)	Ref	NA	NA	Ref	NA	NA
Type TB	Sputum AFB pos.	4,866	63,327	44	69 (51–92)	Ref	NA	NA	Ref	NA	NA
	Bal AFB pos./neg. Cav. PTB	1,109	11,049	11	100 (52–178)	1.2	0.6–2.2	0.66	1.3	0.7–2.5	0.46
	AFB neg. Culture pos. PTB	4,036	50,270	26	52 (35–75)	0.7	0.4–1.2	0.19	0.8	0.5–1.3	0.29
	ETB	5,125	61,968	21	34 (22–51)	0.5	0.3–0.8	0.00	**0.5**	**0.3–0.9**	**0.02**
Drug resistance^d^	(Probably) susceptible ^d^	14,137	174,393	92	53 (43–64)	Ref	NA	NA	Ref	NA	NA
	Mono / Poly H	963	11,838	8	68 (31–128)	1.3	0.6–2.6	0.51	1.4	0.7–2.9	0.38
	Mono / Poly R	36	382	2	524 (88–1730)	9.0	2.2–36.6	0.00	**6.6**	**1.6–27.3**	**0.01**
Comorbidity	No /unknown	13,303	166,034	81	49 (39–60)	Ref	NA	NA	Ref	NA	NA
	Comorbidity^e^	1,213	12,857	12	93 (51–159)	1.7	0.9–3.1	0.10	1.7	0.9–3.2	0.08
	HIV positive	620	7,721	9	117 (57–214)	2.4	1.2–4.7	0.01	1.9	1.0–3.9	0.07
Previous TB episode	No	14,591	180,139	90	50 (40–61)	Ref	NA	NA	Ref	NA	NA
	Yes	545	6,474	12	185 (100–315)	3.6	2.0–6.6	0.00	**3.2**	**1.7–5.9**	**0.00**
Adverse events	Hepatotoxicity	717	9,012	2	22 (4–73)	0.4	0.1–1.6	0.20	NA	NA	NA
	Other	1,043	12,543	9	72 (35–132)	1.3	0.6–2.5	0.49	NA	NA	NA
	No or unknown	13,376	165,057	91	55 (45–68)	Ref	NA	NA	Ref	NA	NA

AFB: acid-fast bacilli; Bal: broncho-alveolar lavage; Cav: cavitary; ETB: extrapulmonary TB; MDR TB: multidrug-resistant TB; Mono/poly H: mono-/poly-resistance against isoniazid other than MDR TB; Mono/poly R: mono-/poly-resistance against rifampicin other than MDR TB; Neg: negative; n: number of patients; Pos: positive; PTB: pulmonary TB; Standard regimen:  2 months isoniazid (H), rifampicin, pyrazinamid (Z), (ethambutol (E))/ 6 months HR(E); TB: tuberculosis.

^a^ Only statistical significant risk factors in the univariate analysis are shown in this table. Univariate values for non-significant variables are provided in the supplementary material.

^b^ Adjusted for sex, treatment regimen, type of TB, drug resistance, comorbidity and previous TB episode.

^c^ Three patients with unknown sex (excluded in the multivariable analysis).

^d^ Until 2005, drug susceptibility was only recorded when drug resistance was detected, hence all cases without registered drug-resistance were categorised as “probably susceptible“.

^e^ Comorbidity such as diabetes, malnutrition, malignancy or use of immunosuppressive medication.

Statistical significant risk factors in the multivariable analysis are shown in bold.

### Risk factors for reinfection

Patients with mono- or poly-resistance against rifampicin (aHR: 11.9; 95% CI: 1.6–88.3) and patients who were experiencing homelessness or undocumented (aHR: 2.7; 95% CI: 1.2–5.8) had a significant higher risk of reinfection. Patients who were born in the Netherlands (aHR: 0.2; 95% CI: 0.1–0.7) or had extrapulmonary TB (adj. HR: 0.2; 95% CI: 0.0–0.6) on the other hand, had a significant lower risk of reinfection (Table 3). In retrospect, the one case with a reinfection with mono- or poly-resistance against rifampicin differed only one repeat in one locus in the VNTR. When repeating the analysis with the less strict definition of reinfection (≥ 2 or more differences in the RFLP or VNTR), the association with both treatment interruption and mono- or poly-resistance against rifampicin disappeared (Supplement Table S5 shows risk factors for reinfection defined as >2 differences in the DNA fingerprint).

**Table 3:** Risk factors^a^ associated with a reinfection, the Netherlands, 1993 to 2016 (n = 15,970)

Category	Level	Cohort n	Person-years follow-up	Events n	Incidence per 100,000 py	Univariate analysis			Multivariable analysis		
						Crude HR	95% CI	p value	aHR^b^	95% CI	p value
Sex^c^	Female	6,451	80,477	9	11	Ref	NA	NA	NA	NA	NA
	Male	9,516	118,376	22	19	1.7	0.8–3.6	0.19	NA	NA	NA
Treatment regimen	Standard regimen	11,686	173,871	30	17	Ref	NA	NA	NA	NA	NA
	Other	4,038	21,473	1	5	0.2	0.0–1.6	0.13	NA	NA	NA
	Unknown	246	3,547	0	0	1.0	0-~	0.97	NA	NA	NA
Treatment outcome	Completed	15,136	186,613	26	14	Ref	NA	NA	Ref.	NA	NA
	Interrupted	834	12,279	5	41	3.2	1.2–8.2	0.02	2.5	1.0–6.7	0.06
DOT	No	12,830	170,991	23	13	Ref	NA	NA	NA	NA	NA
	Yes	3,140	27,900	8	29	1.7	0.8–3.8	0.20	NA	NA	NA
Country of birth and TB incidence^d^	< 100 per 100,000	4,180	51,772	13	25	Ref	NA	NA	Ref	NA	NA
	100–200 per 100,000	2,581	29,526	3	10	0.4	0.1–1.4	0.14	0.4	0.–1.5	0.18
	> 200 per 100,000	4,312	51,491	12	23	0.9	0.4–2.0	0.81	1.3	0.6–3.0	0.49
	The Netherlands	4,731	63,230	3	5	0.2	0.1–0.7	0.01	**0.2**	**0.1–0.7**	**0.01**
	Unknown	166	2,871	0	0	0.0	0-~	0.98	0.0	0-~	0.97
Type TB	Sputum AFB pos.	5,105	66,878	17	25	Ref	NA	NA	Ref	NA	NA
	Bal AFB pos./neg. Cav. PTB	1,151	11,607	2	17	0.5	0.1–2.7	0.66	0.7	0.2–3.0	0.62
	AFB neg. Culture pos. PTB	4,292	53,956	9	17	0.3	0.3–1.4	0.19	0.6	0.3–1.5	0.29
	ETB	5,422	66,451	3	5	0.0	0.0–0.8	0.00	**0.2**	**0.0–0.6**	**0.01**
Drug resistance	(Probably) susceptible^e^	14,905	185,631	28	15	Ref	NA	NA	Ref	NA	NA
	Mono / Poly H	1026	12,824	2	16	1.0	0-24.3	0.98	0.9	0.2–3.9	0.91
	Mono / Poly R	39	436	1	230	13.6	1.8–100.1	**0.01**	**11.9**	**1.6–88.3**	0.02
Homelessness or being undocumented	No	13,666	171,144	21	12	Ref	NA	NA	Ref	NA	NA
	Yes	2,304	27,748	10	36	2.7	1.3–5.8	0.01	**2.7**	**1.2–5.8**	**0.01**
Alcohol or drug use	No	15,322	190,697	28	15	Ref	NA	NA	NA	NA	NA
	Yes	648	8,194	3	37	2.3	0.7–7.5	0.18	NA	NA	NA

AFB: acid-fast bacilli; Cav: cavitary; DOT: directly observed treatment; ETB: extrapulmonary TB; MDR TB: multidrug-resistant TB; mono/poly H: mono/poly resistance against isoniazid other than MDR TB; mono/poly R: mono/poly resistance against rifampicin other than MDR TB; n: number of patients; Neg: negative; Pos: positive; PTB: pulmonary TB; standard regimen: 2 months isoniazid (H), rifampicin, pyrazinamid (Z), (ethambutol (E))/ 6 months HR(E); ref: reference; TB: tuberculosis.

^a^ Only statistically significant risk factors in the univariate analysis are shown in this table. Univariate values for non-significant variables are provided in the supplement material.

^b^ Adjusted for treatment outcome, type of TB, drug resistance, homelessness/being undocumented and country of birth.

^c^ Three patients with unknown sex (excluded in the multivariable analysis).

^d^ TB incidence of country of birth for patients who were born outside the Netherlands.

^e^ Until 2005 drug sensitivity was only recorded when drug resistance was detected, hence all cases without registered drug-resistance were categorised as “probably susceptible“.

Statistical significant risk factors in the multivariate analysis are shown in bold.

## Discussion

The aim of this study was to estimate the rate of reactivation and reinfection of TB patients diagnosed and treated in the Netherlands in the period 1993–2016 after completed and interrupted treatment and to identify risk factors for reactivation and reinfection. The overall rate of reactivation was 55 per 100,000 py in patients who completed their treatment successfully and 318 per 100,000 py for patients who interrupted their treatment. The rate of reinfection was 16 per 100,000 py. In our study we found 0.9% of patients with a reactivation and 0.2% with a reinfection. This is similar to studies from other countries with low TB-incidence for which, although with shorter time frames for follow-up, reactivation rates were reported of 0.3–1.0% [6-9] and reinfection rates of 0.1–0.3% [4,8-20].

Despite the low proportion of patients that developed recurrent TB during the median follow-up period of 15 years, relatively high rates of reactivation were found in the first 5 years after treatment. In the first 2 years after treatment completion, the reactivation rate was 228 per 100,000, while 2−5 years after the completion of TB treatment, the rate of reactivation after treatment completion was still 57 per 100,000. These rates are higher than 50 per 100,000 which is the defined cut-off for a risk group for TB in the Netherlands [23] that should be targeted for active case finding. In particular, patients with sputum smear- or culture-positive pulmonary TB, males, patients with mono or poly drug-resistance against rifampicin and patients who experienced a previous TB episode had a high incidence of reactivation. Presently, ex-TB patients are not considered as a risk group in the Netherlands and not targeted for further screening or follow-up [23]. In view of our findings, this should be reconsidered, at least for patients with risk factors for recurrence. Among interrupters, in the first 2 years after treatment cessation the reactivation rate is extremely high with a rate of 1,798 per 100,000 py; it is evident that this group is most important to find and restart treatment.

The proportion of patients presenting reactivation by timing of treatment interruption are quite similar between those who were treated less than 3 months and those treated less than 6 months (Table 1). This was unexpected and is possibly because the registered duration of treatment did not reflect if dosages were taken each day, as this is not captured in the registration. As expected, we note a smaller proportion of reactivation in patients receiving treatment for at least 7 months. With respect to risk factors for reactivation and reinfection, we showed that males had a higher risk of reactivation after completing treatment, which is also observed in other studies [9,11,24,25]. The WHO has reported a male to female ratio of 1.7:1 among global TB notifications in 2015 [26]. This can explain the higher risk of reactivation in males in the current study.

Among patients who interrupted their treatment in the first episode, patients who received medication under DOT and were undocumented migrants or people experiencing homelessness had a higher risk of reactivation. A possible explanation for this finding is the fact that all patients get treatment support from dedicated TB nurses, but DOT is offered to selective patients with a perceived high risk to be non-adherent to treatment, patients who have recurrent TB or patients with multidrug-resistant (MDR) TB [23]. Those who interrupt TB treatment despite daily supervision and intensive support are likely to have been more non-adherent than other patients. This was also observed in the US [10]. This study confirms need for intensive patient support given on daily basis, to reduce the risk for recurrent TB.

Several studies found a strong association between reactivation of TB and drug resistance, especially multidrug resistance and isoniazid resistance [10,14,17]. We excluded MDR TB patients from our cohort analysis because they are treated with individualised regimens and of note, we did not find any recurrent cases among the excluded patients with MDR TB. We did not find an association of isoniazid mono- or poly-resistance with reactivation and/or reinfection. However, we did find that patients with mono/poly rifampicin resistance had a higher risk of reactivation after completing treatment and of reinfection. The three patients with recurrent rifampicin-resistant TB in our cohort were diagnosed initially before 2006 and all received a standard TB regimen. This may explain the high reactivation rate. Since 2007, patients with mono or poly rifampicin-resistant TB disease in the Netherlands are treated with an appropriate regimen containing second-line anti-TB drugs, based on the sensitivity pattern of the isolated strain as recommended by national and international guidelines [23,27].

The association of mono or poly rifampicin-resistant TB with reinfection can be explained by a too-strict definition of reinfection (≥ 1 loci difference in fingerprint results). When we repeated the analysis defining reinfection as more than two differences in the RFLP or VNTR, the association of rifampicin resistance with reinfection disappeared. After all, genetic drift does appear in some of the strains and finding a difference of one or even more than one VNTR loci may still mark infection by the same strain [28]. The strict definition of reinfection may have caused misclassification of reactivation. When we redefined five cases with potential misclassification of reinfection into reactivation and repeated the risk analysis for reactivation, we found the same associations with the risk for reactivation and the association with HIV infection became stronger (Supplement Table S4).

Several studies have reported a higher rate of recurrent TB among patients with HIV [10,14]. Reduced immunity due to the reduction of CD4 + T-cells may explain the higher risk of recurrent TB in patients with an HIV co-infection [28]. Studies from countries with a low incidence of TB, England and Wales, Spain and the US, also found an association between HIV and recurrent TB, but they did not distinguish between a reactivation and reinfection [10,29,30]. This is the first study in a low TB incidence country that studied the association between HIV, other comorbidity and reactivation. We found a significant association when we used the less strict definition of reactivation.

Patients born in the Netherlands had a lower risk of reinfection in this study. This reflects the infection pressure typical to the TB epidemic in the Netherlands. In the Netherlands, the TB incidence among foreign-born residents is more than 20 times higher than among the native population [23]. It has been shown that migrants from high endemic countries have a higher risk of TB in their host country for at least 10 years after arrival [31]. We therefore assume that the increased risk of reinfection is attributable to a high transmission in this population group.

Extrapulmonary TB was associated with a lower risk of both reactivation and reinfection. Previous studies have also found a similar association but did not differentiate between a reactivation and reinfection [4,6-8]. The lower risk of reinfection among patients with extrapulmonary TB was neither described before nor explained in the literature. Therefore, more studies are needed to confirm and explain this association. However, extrapulmonary TB is known as a milder and more paucibacillary form of TB, what makes the disease easier to treat [32]. Preliminary data from a study into shorter treatment for minimal TB in children (SHINE trial) have shown that in children with non-severe TB, a standard TB treatment regimen with a continuation phase of 4 months is equivalent to a shorter regimen with a continuation phase of 2 months [33]. This could explain also why a lower risk of reactivation is found in adult patients with extrapulmonary TB. Our study showed that even after interrupting treatment, only 2% of the patients with extrapulmonary TB had a reactivation compared with patients with pulmonary TB (6%) (Supplement Table S3). This suggests that for patients with mild forms of extrapulmonary TB, shorter treatment regimens could be considered.

Our study had some limitations. Firstly, there was likely an underestimation of the patients with recurrent TB because we did not actively follow the patients included in the cohort. Therefore, we do not know if patients have died, or have left the country between the first and second episode of disease. Moreover, TB disease in patients with recurrent TB is not always confirmed with culture. We included only confirmed culture-positive patients with available DNA fingerprint data, which might have caused an underestimation of the reactivation rate. The low number of recurrent events may have resulted in unstable estimates and wide confidence intervals for the risk analysis for some of the factors explored. Nevertheless, strengths of this study are that it had a much larger follow-up period (maximum of 24 years) compared with previous studies (maximum of 18 years) [13] and larger cohort with available DNA fingerprint data in the first and second episode of the disease compared with other studies where the maximum was 8,084 patients [13]. This made it possible to determine risk factors for both a reactivation and reinfection.

## Conclusions

In conclusion, during the first 2 years after completion of TB treatment reactivation rates are higher than 200 per 100,000 py and 65 times higher in patients who interrupt their treatment. Informing patients about the risk of recurrence and regular monitoring and follow-up of those with a higher risk of recurrence is important, at least in the first 2─5 years after treatment. Studies into shortening the TB treatment in the milder form of (extrapulmonary) TB should be undertaken to reduce the burden of treatment in the patient.

## Supplementary Data

396000 Supplementary Material
