# Supplementary material for: Recurrent tuberculosis in the Netherlands – a 24-year follow-up study, 1993 to 2016
Source: Euro Surveill. 2022 Mar 24;27(12):2100183. doi: 10.2807/1560-7917.ES.2022.27.12.2100183 (PMC8950855; doi:10.2807/1560-7917.ES.2022.27.12.2100183)
Supplement: Supplementary Material [file 21-00183_ERKENS_TB_SupplementaryMaterial.pdf]

**Supplementary Tables for paper: 'Recurrent tuberculosis in the Netherlands – a 24 year follow-up study'.**

This supplementary material is hosted by *Eurosurveillance* as supporting information alongside the article 'Recurrent tuberculosis in the Netherlands – a 24 year follow-up study' on behalf of the authors, who remain responsible for the accuracy and appropriateness of the content. The same standards for ethics, copyright, attributions and permissions as for the article apply. Supplements are not edited by *Eurosurveillance* and the journal is not responsible for the maintenance of any links or email addresses provided therein.

This Supplement has 3 parts:

1. Calculations of risk rates per follow-up time (Tables S1 and S2)
2. Risk factors associated with a reactivation for patients who interrupted their treatment (Table S3)
3. Sensitivity analysis using reactivation defined as ≤2 bands difference in the RFLP or ≤2 repeats difference in the VNTR. (Table S4) and reinfection defined as >2 bands difference in the RFLP or >2 repeats difference in the VNTR (Table S5).

**Part 1. Calculations of risk rates per follow-up time**

**Table S1. Incidence rates for reactivation, reinfection and recurrence.**

| | | N | Sum py | Event | Rate per 100,000 py | Rate ratio | | Time to event in years | |
|---|---|---|---|---|---|---|---|---|---|
| | | | | | | Ratio | 95% CI | Median | Q1-Q3 |
| Reactivation | completers | 15,136 | 186,613 | 102 | 55 | | | 1.3 | 0.6-2.9 |
| | interrupters | 834 | 12,279 | 39 | 318 | 5.8 | 4.0-8.4 | 0.9 | 0.5-2.0 |
| Total reactivation | | 15,970 | 198,891 | 141 | 71 | | | 1.1 | 0.6-2.7 |
| Reinfection | | 15,970 | 198,891 | 31 | 16 | | | 5.5 | 2.1-8.4 |

CI=confidence interval, completers=patients who completed their treatment in the first episode, interrupters=patients who interrupted treatment in the first episode, py=person years, Q1=first quartile, Q3=third quartile.

**Table S2. Incidence rates for reactivation, reinfection and recurrence by duration of follow-up. (underlying data for figure 1)**

| | | <2 yrs. of follow-up | | | | 2-5 yrs. of follow-up | | | | >5 yrs. of follow-up | | | |
|---|---|---|---|---|---|---|---|---|---|---|---|---|---|
| | Cohort (n) | Py follow-up | Events (n) | Incidence per 100,000 py | 95% CI | Py follow-up | Events (n) | Incidence per 100,000 py | 95% CI | Py follow-up | Events (n) | Incidence per 100,000 py | 95% CI |
| Reactivation | 15,970 | 30,998 | 96 | 310 | 252-377 | 42,560 | 33 | 78 | 54-108 | 125,582 | 12 | 10 | 5-16 |
| Reactivation completers | 15,136 | 29,386 | 67 | 228 | 178-288 | 40,243 | 23 | 57 | 37-84 | 117,204 | 12 | 10 | 6-17 |
| Reactivation interrupters | 834 | 1,613 | 29 | 1,798 | 1,227-2,548 | 2,317 | 10 | 432 | 219-769 | 8,378 | - | - | - |
| Reinfection | 15,970 | 30,998 | 6 | 19 | 8-40 | 42,560 | 9 | 21 | 10-39 | 125,582 | 16 | 13 | 8-20 |
| Recurrence (total)# | 15,970 | 30,998 | 102 | 329 | 270-398 | 42,560 | 42 | 99 | 72-132 | 125,582 | 28 | 22 | 15-32 |

CI=confidence interval, completers=patients who completed their treatment in the first episode, interrupters=patients who interrupted treatment in the first episode, n=number of patients, py=person years, #=after completing and interrupting treatment.

## Part 2. Risk factors associated with a reactivation for patients who interrupted their treatment.

### Table S3. Significant risk factors associated with a reactivation for patients who interrupted their treatment (N= 834)

| | | | | | | Univariate analysis | | Multivariable analysis | | |
|---|---|---|---|---|---|---|---|---|---|---|
| Category | Level | Cohort (N) | Person-years follow-up | events (n) | Incidence per 100,000 py | HR Unadj. | p-value | HR adj. | 95% CI[1] | p-value |
| Sex | Females | 289 | 4,279 | 14 | 327 | Ref | | | | |
| | Males | 545 | 8,000 | 25 | 313 | 1.0 | 0.88 | | | |
| Treatment regimen | Standard regimen | 600 | 9,490 | 31 | 327 | Ref | | | | |
| | Other | 164 | 1,639 | 6 | 366 | 0.8 | 0.52 | | | |
| | Unknown | 70 | 1,150 | 2 | 174 | 0.5 | 0.41 | | | |
| DOT | No | 645 | 10,361 | 21 | 203 | Ref | | Ref | | |
| | Yes | 189 | 1,918 | 18 | 939 | 3.2 | 0.00 | 2.2 | 1.1-4.3 | 0.02 |
| Country of birth | <100 per 100.000 | 244 | 3,545 | 15 | 423 | Ref | | | | |
| | 100-200 per 100.000 | 135 | 1,845 | 8 | 434 | 1.0 | 0.91 | | | |
| | >200 per 100.000 | 246 | 3,833 | 6 | 157 | 0.4 | 0.05 | | | |
| | Netherlands | 192 | 2,764 | 10 | 362 | 0.8 | 0.66 | | | |
| | Onbekend | 17 | 292 | 0 | 0 | 0.0 | 0.97 | | | |
| Age group | 0-14 | 18 | 270 | 0 | 0 | 0.0 | 0.97 | | | |
| | 15-24 | 202 | 3,086 | 7 | 227 | 0.9 | 0.92 | | | |
| | 25-34 | 248 | 3,719 | 11 | 296 | 1.2 | 0.73 | | | |
| | 35-44 | 147 | 2,150 | 12 | 558 | 2.3 | 0.15 | | | |
| | 45-54 | 66 | 886 | 2 | 226 | 0.8 | 0.82 | | | |
| | 55-64 | 44 | 639 | 3 | 470 | 1.9 | 0.39 | | | |
| | 65+ | 109 | 1,529 | 4 | 262 | Ref | | | | |
| Type of TB | Sputum AFB pos. | 239 | 3,551 | 17 | 479 | Ref | | Ref | | |
| | Bal AFB pos./neg. Cav. PTB | 42 | 559 | 3 | 537 | 1.0 | 0.99 | 1.1 | 0.3-3.8 | 0.88 |
| | AFB neg. Culture pos. PTB | 256 | 3,686 | 13 | 353 | 0.7 | 0.32 | 0.7 | 0.3-1.4 | 0.30 |
| | ETB | 297 | 4,483 | 6 | 134 | 0.3 | 0.01 | **0.4** | **0.1-1.0** | 0.06 |
| Drug resistance | (Probably) susceptible# | 768 | 11,238 | 38 | 338 | Ref | | | | |
| | Mono / Poly H | 63 | 987 | 1 | 101 | 0.3 | 0.25 | | | |
| | Mono / Poly R | 3 | 54 | 0 | 0 | 0.0 | 0.98 | | | |
| Homeless/ undocumented | No | 650 | 9,844 | 21 | 213 | Ref | | Ref | | |
| | Yes | 184 | 2,435 | 18 | 739 | 3.2 | 0.00 | 2.1 | 1.1-4.1 | 0.03 |
| Alcohol/ Drugs abuse | No | 768 | 11,404 | 30 | 263 | Ref | | | | |
| | Yes | 66 | 875 | 9 | 1029 | 3.7 | 0.00 | | | |
| Comorbidity | No /unknown | 733 | 10,867 | 33 | 304 | Ref | | | | |
| | Comoribidity | 53 | 657 | 4 | 609 | 1.7 | 0.32 | | | |
| | HIV positive | 48 | 755 | 2 | 265 | 0.9 | 0.92 | | | |
| Previous TB episode [2] | No | 792 | 11,662 | 36 | 309 | Ref | | | | |
| | Yes | 42 | 617 | 3 | 487 | 1.6 | 0.44 | | | |
| Adverse events | Hepatotoxicity | 69 | 986 | 2 | 203 | 0.6 | 0.49 | | | |
| | Other | 138 | 1,938 | 7 | 361 | 1.0 | 0.91 | | | |

| | | | | | | | | | | |
|---|---|---|---|---|---|---|---|---|---|---|
| | No or unknown | 627 | 9,355 | 30 | 321 | Ref | | | | |
| Treatment duration | 0-3 months | 305 | 4,419 | 16 | 362 | Ref | | Ref | | |
| | 4-6 months | 291 | 3,824 | 19 | 497 | 1.3 | 0.50 | 1.0 | 0.5-2.0 | 0.97 |
| | ≥7 months | 238 | 4,036 | 4 | 99 | 0.3 | 0.04 | **0.3** | **0.1-0.9** | **0.03** |

GREY = Variable not included in initial model multivariable Cox regression analysis

[1]= Adjusted for treatment in months, DOT, type of TB and homeless/undocumented. **Statistical significant risk factors in the multivariable analysis are shown in bold.**

[2]= previous TB episode, i.e. a disease episode before this register started (before 1993) or outside the Netherlands

AFB= acid-fast bacilli, Bal = broncho-alveolar lavage, Cav = cavitary, ETB= extra-pulmonary TB, N= number of patients, Neg= negative, Periph= peripheral, Pos= positive, PTB= pulmonary TB. Mono/poly H= mono-/poly resistance against isoniazid other than MDR TB, mono/poly R= mono-/poly resistance against rifampicin other than MDR TB, N= number of patients, Neg= negative, Pos= positive, PTB= pulmonary TB, Standard regimen= 2HRZ(E)/6HR(E). # Until 2005 drug sensitivity was only recorded when drug resistance was detected, hence all cases without registered drug-resistance were categorized as “probably sensitive“.

**Part 3. Sensitivity analysis using reactivation defined as ≤2 bands difference in the RFLP of ≤2 repeats difference in the VNTR.**

**Table S4. Significant risk factors associated with a reactivation for patients who completed their treatment (N=15,136) with reactivation defined as less than 2 differences in the DNA-fingerprint**

| | | | | | | Univariate analysis reinfection | | | Multivariable analysis reinfection | | |
|---|---|---|---|---|---|---|---|---|---|---|---|
| Category | Level | Cohort (N) | Person-years follow-up | events (n) | Incidence per 100,000 py | HR Unadj. | 95% CI | p-value | HR adj. | 95% CI[1] | p-value |
| Sex | Females | 6,349 | 77,157 | 28 | 36 | Ref | | | Ref | | |
| | Males | 9,225 | 111,581 | 78 | 70 | 1.9 | 1.3-3.0 | 0.00 | 1.7 | 1.1=2.7 | 0.01 |
| Treatment regimen | Standard regimen | 11,162 | 165,387 | 86 | 52 | Ref | | | Ref | | |
| | Other | 4,234 | 20,931 | 15 | 72 | 0.6 | 0.4-1.1 | 0.12 | 0.6 | 0.4-1.1 | 0.10 |
| | Unknown | 181 | 2,459 | 5 | 203 | 3.6 | 1.5-8.9 | 0.01 | 3.8 | 1.5-9.4 | 0.00 |
| DOT | No | 12,457 | 161,879 | 83 | 51 | Ref | | | | | |
| | Yes | 3,120 | 26,897 | 23 | 86 | 1.2 | 0.7-1.9 | 0.47 | | | |
| Country of birth | <100 per 100.000 | 4079 | 48,966 | 25 | 51 | Ref | | | | | |
| | 100-200 per 100.000 | 2528 | 28,007 | 21 | 75 | 1.4 | 0.8-2.4 | 0.29 | | | |
| | >200 per 100.000 | 4191 | 48,415 | 24 | 50 | 0.9 | 1.5-1.6 | 0.80 | | | |
| | Nederland | 4629 | 60,799 | 36 | 59 | 1.2 | 0.7-2.0 | 0.43 | | | |
| | Onbekend | 150 | 2,590 | 0 | 0 | 0.0 | 0-~ | 0.94 | | | |
| Age group | 0-14 | 460 | 6,000 | 4 | 67 | 1.0 | 0.3-3.0 | 0.97 | | | |
| | 15-24 | 3078 | 38,520 | 16 | 42 | 0.6 | 0.3-1.2 | 0.16 | | | |
| | 25-34 | 4282 | 53,022 | 20 | 38 | 0.6 | 0.3-1.1 | 0.07 | | | |
| | 35-44 | 2704 | 32,026 | 22 | 69 | 1.0 | 0.5-1.8 | 0.94 | | | |
| | 45-54 | 1784 | 20,261 | 16 | 79 | 1.1 | 0.6-2.2 | 0.80 | | | |
| | 55-64 | 1237 | 14,263 | 11 | 77 | 1.1 | 0.5-2.3 | 0.86 | | | |
| | 65+ | 2032 | 24,685 | 17 | 69 | Ref | | | | | |

| | | | | | | | | | | | |
|---|---|---|---|---|---|---|---|---|---|---|---|
| Type TB | Sputum AFB pos. | 4,967 | 64,095 | 45 | 70 | Ref | | | Ref | | |
| | Bal AFB pos./neg. Cav. PTB | 1141 | 11,166 | 13 | 116 | 1.4 | 0.7-2.5 | 0.33 | 1.5 | 0.8-2.7 | 0.23 |
| | AFB neg. Culture pos. PTB | 4,153 | 50,782 | 27 | 53 | 0.7 | 0.5-1.2 | 0.21 | 0.8 | 0.5-1.3 | 0.33 |
| | ETB | 5,316 | 62,733 | 21 | 33 | 0.4 | 0.3-0.8 | 0.00 | **0.5** | **0.3-0.9** | **0.02** |
| Drug resistance | (Probably) susceptible# | 14,249 | 174,396 | 94 | 54 | Ref | | | Ref | | |
| | Mono / Poly H | 970 | 11,838 | 9 | 76 | 1.4 | 0.7-2.8 | 0.33 | 1.5 | 0.8-3.1 | 0.22 |
| | Mono / Poly R | 36 | 382 | 3 | 786 | 13.3 | 4.2-40.0 | 0.00 | **8.7** | **2.7-28.4** | **0.00** |
| Homeless/ undocumented | No | 13,409 | 163,262 | 88 | 54 | Ref | | | | | |
| | Yes | 2168 | 25,515 | 18 | 71 | 1.3 | 0.8-2.1 | 0.37 | | | |
| Alcohol/ Drugs abuse | No | 14,989 | 181,422 | 102 | 56 | Ref | | | | | |
| | Yes | 588 | 7,354 | 4 | 54 | 1.0 | 0.4-2.7 | 0.96 | | | |
| Comorbidity | No /unknown | 13,654 | 167,819 | 84 | 50 | Ref | | | Ref | | |
| | Comoribidity | 1285 | 13,125 | 12 | 91 | 1.6 | 0.9-2.9 | 0.13 | 1.6 | 0.9-3.0 | 0.12 |
| | HIV positive | 638 | 7,833 | 10 | 128 | 2.5 | 1.3-4.9 | 0.01 | **2.0** | **1.0-3.9** | **0.04** |
| Previous TB episode [2] | No | 14,994 | 182,020 | 94 | 52 | Ref | | | | | |
| | Yes | 583 | 6,757 | 12 | 178 | 3.6 | 1.8-6.0 | 0.00 | **3.0** | **1.6-5.5** | **0.00** |
| Adverse events | Hepatotoxicity | 735 | 9,109 | 2 | 22 | 0.4 | 0.1-1.6 | 0.19 | | | |
| | Other | 1104 | 12,965 | 10 | 77 | 1.3 | 0.7-2.6 | 0.39 | | | |
| | No or unknown | 13738 | 166,703 | 94 | 56 | Ref | | | | | |

GREY = Variable not included in initial model multivariable Cox regression analysis

[1]= Adjusted for treatment in months, DOT, type of TB and homeless/undocumented. **Statistical significant risk factors in the multivariate analysis are shown in bold.**

[2]= previous TB episode, i.e. a disease episode before this register started (before 1993) or outside the Netherlands

AFB= acid-fast bacilli, Bal = broncho-alveolar lavage, Cav = cavitary, ETB= extra-pulmonary TB, N= number of patients, Neg= negative, Periph= peripheral, Pos= positive, PTB= pulmonary TB. Mono/poly H= mono-/poly resistance against isoniazid other than MDR TB, mono/poly R= mono-/poly resistance against rifampicin other than MDR TB, N= number of patients, Neg= negative, Pos= positive, PTB= pulmonary TB, Standard regimen= 2HRZ(E)/6HR(E). # Until 2005 drug sensitivity was only recorded when drug resistance was detected, hence all cases without registered drug-resistance were categorized as “probably sensitive“.

**Table S5. Significant risk factors associated with a reinfection (N=15,970) with reinfection defined as >2 differences in the DNA-fingerprint**

| | | | | | | Univariate analysis | | | Multivariable analysis | | |
|---|---|---|---|---|---|---|---|---|---|---|---|
| Category | Level | Cohort (N) | Person-years follow-up | events (n) | Incidence per 100,000 py | HR Unadj. | 95% CI | p-value | HR adj. | 95% CI[1] | p-value |
| Sex | Females | 6,648 | 81,515 | 7 | 9 | Ref | | | | | |
| | Males | 9,802 | 119,870 | 19 | 16 | 1.9 | 0.8-4.4 | 0.16 | | | |
| Treatment regimen | Standard regimen | 11,772 | 175,050 | 26 | 15 | Ref | | | | | |
| | Other | 4,426 | 22,700 | 0 | 0 | 0.0 | 0-5.1 | 0.19 | | | |
| | Unknown | 255 | 3,673 | 0 | 0 | 0.0 | 0-~ | 0.63 | | | |
| Treatment outcome | Completed | 15136 | 186,613 | 26 | 14 | Ref | | | Ref | | |
| | Interrupted | 834 | 12,279 | 5 | 41 | 3.0 | 1.0-8.6 | 0.05 | 2.5 | 0.9-7.3 | 0.10 |
| DOT | No | 13,126 | 172,488 | 20 | 12 | Ref | | | | | |
| | Yes | 3,327 | 28,936 | 6 | 21 | 1.4 | 0.6-3.6 | 0.45 | | | |
| Country of birth | <100 per 100.000 | 4334 | 52,642 | 11 | 21 | Ref | | | Ref | | |
| | 100-200 per 100.000 | 2676 | 29,980 | 1 | 3 | 0.2 | 0.0-1.2 | 0.07 | 0.4 | 0.1-1.5 | 0.18 |
| | >200 per 100.000 | 4443 | 52,286 | 11 | 21 | 1.0 | 0.4-2.3 | 0.97 | 1.3 | 0.6-3.0 | 0.49 |
| | Netherlands | 4833 | 63,633 | 3 | 5 | 0.2 | 0.1-0.8 | 0.02 | **0.2** | **0.1-0.7** | **0.01** |
| | Unknown | 167 | 2,882 | 0 | 0 | 0.0 | 0-~ | 0.97 | 0.0 | 0-~ | 0.97 |
| Age group | 0-14 | 478 | 6,271 | 2 | 32 | Ref | | | | | |
| | 15-24 | 3290 | 41,703 | 9 | 22 | 0.7 | 0.1-3.1 | 0.60 | | | |
| | 25-34 | 4544 | 56,898 | 8 | 14 | 0.4 | 0.1-2.0 | 0.29 | | | |
| | 35-44 | 2856 | 34,223 | 3 | 9 | 0.3 | 0.0-1.6 | 0.14 | | | |
| | 45-54 | 1854 | 21,157 | 3 | 14 | 0.4 | 0.1-2.5 | 0.34 | | | |
| | 55-64 | 1283 | 14,910 | 1 | 7 | 0.2 | 0.0-2.2 | 0.19 | | | |
| | 65+ | 2148 | 26,262 | 0 | 0 | 0.0 | 0-~ | 0.97 | | | |
| Type TB | Sputum AFB pos. | 5,218 | 67,767 | 15 | 22 | Ref | | | Ref | | |
| | Bal AFB pos./neg. Cav. PTB | 1185 | 11,726 | 0 | 0 | 0.0 | 0-~ | 0.97 | 0.0 | 0-~ | 0.97 |

| | | | | | | | | | | | |
|---|---|---|---|---|---|---|---|---|---|---|---|
| | AFB neg. Culture pos. PTB | 4,424 | 54,619 | 8 | 15 | 0.7 | 0.3-1.6 | 0.34 | 0.6 | 0.3-1.5 | 0.30 |
| | ETB | 5,626 | 67,312 | 3 | 4 | 0.2 | 0.1-0.7 | 0.01 | **0.2** | **0.1-0.7** | **0.01** |
| Drug resistance | (Probably) susceptible# | 15,022 | 185,634 | 25 | 13 | Ref | | | | | |
| | Mono / Poly H | 1033 | 12,825 | 1 | 8 | 0.6 | 0.8-4.3 | 0.59 | | | |
| | Mono / Poly R | 39 | 436 | 0 | 0 | 0.0 | 0-~ | 0.99 | | | |
| Homeless/ undocumented | No | 14,090 | 173,403 | 17 | 10 | Ref | | | Ref | | |
| | Yes | 2363 | 28,021 | 9 | 32 | 3.2 | 1.4-7.1 | 0.01 | **3.0** | **1.3-6.9** | **0.01** |
| Alcohol/ Drugs abuse | No | 15,794 | 193,177 | 24 | 12 | Ref | | | | | |
| | Yes | 659 | 8,247 | 2 | 24 | 1.9 | 0.5-8.2 | 0.36 | | | |
| Comorbidity | No /unknown | 14,421 | 179,009 | 24 | 13 | Ref | | | | | |
| | Comoribidity | 1344 | 13,814 | 0 | 0 | 0.0 | 0-~ | 0.97 | | | |
| | HIV positive | 688 | 8,601 | 2 | 23 | 1.7 | 0.4-7.2 | 0.47 | | | |
| Previous TB episode [2] | No | 15,820 | 193,948 | 26 | 13 | Ref | | | | | |
| | Yes | 633 | 7,476 | 0 | 0 | 0.0 | 0.0-381.6 | 0.51 | | | |
| Adverse events | Hepatotoxicity | 806 | 10,095 | 1 | 10 | 0.9 | 0.1-5.8 | 0.96 | | | |
| | Other | 1254 | 14,996 | 3 | 20 | 0.5 | 0.5-5.3 | 0.49 | | | |
| | No or unknown | 14393 | 176,333 | 22 | 12 | Ref | | | | | |
| Treatment duration | 0-3 months | 318 | 4,543 | 1 | 22 | Ref | | | | | |
| | 4-6 months | 1,431 | 17,032 | 4 | 23 | 0.9 | 0.1-8.4 | 0.96 | | | |
| | ≥7 months | 14704 | 179,849 | 21 | 12 | 0.5 | 0.1-3.6 | 0.49 | | | |

GREY = Variable not included in initial model multivariable Cox regression analysis

[1]= Adjusted for treatment in months, DOT, type of TB and homeless/undocumented. **Statistical significant risk factors in the multivariate analysis are shown in bold.**

[2]= previous TB episode, i.e. a disease episode before this register started (before 1993) or outside the Netherlands

AFB= acid-fast bacilli, Bal = broncho-alveolar lavage, Cav = cavitary, ETB= extra-pulmonary TB, N= number of patients, Neg= negative, Periph= peripheral, Pos= positive, PTB= pulmonary TB. Mono/poly H= mono-/poly resistance against isoniazid other than MDR TB, mono/poly R= mono-/poly resistance against rifampicin other than MDR TB, N= number of patients, Neg= negative, Pos= positive, PTB= pulmonary TB, Standard regimen= 2HRZ(E)/6HR(E). # Until 2005 drug sensitivity was only recorded when drug resistance was detected, hence all cases without registered drug-resistance were categorized as “probably sensitive“.
